# DNA methylation changes in infants between 6 and 52 weeks

**DOI:** 10.1038/s41598-019-54355-z

**Published:** 2019-11-26

**Authors:** Ellen Wikenius, Vibeke Moe, Lars Smith, Einar R. Heiervang, Anders Berglund

**Affiliations:** 1 H. Lee Moffitt Cancer Center & Research Institute, Tampa, Florida USA; 2 Institute of Clinical Medicine, Faculty of Medicine, University of Oslo, Oslo, Norway; 3 Department of Psychology, Faculty of Social Sciences, University of Oslo, Oslo, Norway; 4 The Center for Child and Adolescent Mental Health, Eastern and Southern Norway (RBUP), Oslo, Norway; 5 Oslo University Hospital, Oslo, Norway

**Keywords:** DNA methylation, Paediatric research

## Abstract

Infants undergo extensive developments during their first year of life. Although the biological mechanisms involved are not yet fully understood, changes in the DNA methylation in mammals are believed to play a key role. This study was designed to investigate changes in infant DNA methylation that occurs between 6 and 52 weeks. A total of 214 infant saliva samples from 6 or 52 weeks were assessed using principal component analyses and t-distributed stochastic neighbor-embedding algorithms. Between the two time points, there were clear differences in DNA methylation. To further investigate these findings, paired two-sided student’s t-tests were performed. Differently methylated regions were defined as at least two consecutive probes that showed significant differences, with a q-value < 0.01 and a mean difference > 0.2. After correcting for false discovery rates, changes in the DNA methylation levels were found in 42 genes. Of these, 36 genes showed increased and six decreased DNA methylation. The overall DNA methylation changes indicated decreased gene expression. This was surprising because infants undergo such profound developments during their first year of life. The results were evaluated by taking into consideration the extensive development that occurs during pregnancy. During the first year of life, infants have an overall three-fold increase in weight, while the fetus develops from a single cell into a viable infant in 9 months, with an 875-million-fold increase in weight. It is possible that the findings represent a biological slowing mechanism in response to extensive fetal development. In conclusion, our study provides evidence of DNA methylation changes during the first year of life, representing a possible biological slowing mechanism. We encourage future studies of DNA methylation changes in infants to replicate the findings by using a repeated measures model and less stringent criteria to see if the same genes can be found, as well as investigating whether other genes are involved in development during this period.

## Introduction

In the first year of life, profound changes take place in human physical and neurodevelopmental functioning^1^. Infants learn to sit, stand up, walk, and say their first words^2^. This rapid growth occurs in an orderly and regulated sequence, laying the building blocks for future growth^1^. Although infant developmental milestones are well-known^2^, the underlying biological mechanisms driving this development in the first year of life are not yet fully understood. Epigenetics is believed to play an important role in mediating the developmental changes in mammalian development^3^, but to the best of our knowledge, few studies have been published specifically on the epigenetic role in infant development.

Here, DNA methylation is one type of epigenetic mechanism that regulates gene expression without altering the DNA sequence^4^; it is involved in many cellular processes and is known to be relatively stable. However, DNA methylation might change within an individual over time^5^. Embryonic and pluripotent stem cell DNA methylation is close to zero^6^ but changes extensively from fertilization to implantation^7^. Children from 2 to 16 years of age have been found to have increased levels of the age-related gene DNA methylation, with the greatest changes being found early, from 2 to 10 years of age^8^.

Two age-specific epigenetic occurrences have been studied: epigenetic drift^9^ and the epigenetic clock^6,10^. Epigenetic drift relates to the changes in DNA methylation over time, which differs among individuals^11^. Examples can be found in twin DNA methylation studies that find that DNA methylation differences increase over time in relation to age and different lifestyles^9,12^. The epigenetic clock, which was conceived by Horvath^6^ and Hannum^13^, is a means to calculate a person’s epigenetic age based on DNA methylation calculated from age-related CpG sites. Studies of age-related DNA methylation changes have mainly been of older populations, which are confounded by decade-long processes of environmental age-effecting exposures and aging itself; studies of younger populations have therefore been encouraged^14^.

Gene function and its epigenetic regulation are far from completely understood^15^, but DNA methylation has been associated with the regulation of gene expression^16^, and increases in DNA methylation have been associated with decreased gene expression^17^ although it depends on where the DNA methylation occurs in the gene. Gene DNA methylation can be loosely divided into a few different regions: the promoter regions (TSS1500 and TSS200), 5′ untranslated region (UTR), the 1st exon, the gene body, and 3′UTR. It is thought that the promoter regions initiate the transcription of a particular gene^16^, and it is widely recognized that the DNA methylation of this area is associated with decreased gene expression^17^.

To the best of our knowledge, there have been no epigenome-wide association studies (EWAS) of DNA methylation changes in infants during the first year of life. Hence, the objectives of the current study were three-fold: (1) to assess whether DNA methylation changes between 6 and 52 weeks (2), and if so, to describe the genes associated with these DNA methylation changes, and (3) to discuss the findings in association with infant development.

## Materials and Methods

### Ethical approval

This study was approved by the Regional Committees for Medical and Health Research Ethics (REK) in Norway (REK reference number: 2011/560/REK). Informed consent was obtained for infant participation from all mothers. The experiments were performed in accordance with relevant guidelines and regulations.

### Participants

A subsample of 172 infants from the “Little in Norway” study^18^ was used for the current study; for the experiment, 274 saliva samples from infants 6 weeks old (n = 62), 52 weeks old (n = 30), or both (n = 61) were selected. The sociodemographic variables are shown in Table 1. The saliva samples were collected using the Oragene DNA assisted collection kit (OG-575) (DNA Genotek, 2018). Quality control (QC) and cell composition analyses removed 60 saliva samples, leaving 214 saliva samples from 153 infants for epigenetic analyses. The infants’ mothers volunteered information about their age, education level, and marital status. Data on fetal gender were collected from birth records.

**Table 1:** Sociodemographic variables

At birth (N = 153)	Mean (SD)
**Maternal age**	30 years (5 years)
Gestational age	40 weeks (1 week)
Weight	3,621 g (476 g)
	**Percentage**
Girls	72 (47%)
Boys	81 (53%)
**Maternal completed education**	
Primary education	2 (1%)
Secondary education	34 (22%)
College	53 (35%)
University	64 (42%)
**Maternal marital status**	
Living with partner	97 (63%)
Married	49 (32%)
Single	5 (3%)
Other	2 (3%)

### Saliva samples and DNA methylation profiling

The 274 infant saliva samples were collected at 6 and 52 weeks using the OG-575 assisted collection kit. DNA methylation profiling was conducted at the Norwegian Sequencing Centre using the Infinium Human Methylation 450 K BeadChip array (Illumina, San Diego, CA, USA). DNA extraction was conducted using the Oragene prep-IT.L2P kit (DNA Genotek, Ottawa, ON, Canada), and the quantity was assessed using PicoGreen (Thermo Fisher, Waltham, MA, USA). The EZ-96 DNA Methylation-Gold Kit (Zymo Research) was used for the bisulfite conversion of 320–500 ng of the saliva DNA samples.

The DNA samples were randomly located on 96-well plates to minimize potential batch effects, and beta-mixture quantile normalization (BMIQ) was used to normalize the β-values^19^. During QC, 29,233 cross-reactive probes^20^, 4,232 probes with single nucleotide polymorphisms (SNPs) at the CpG site, 16,819 probes and 13 samples with unreliable measurements (detection p-values > 0.01), 9,675 probes located on the sex chromosomes, and 2,303 non-CpG probes were removed. In total, 18 samples were removed, leaving 256 for cell composition assessment.

### Cell composition

Because the human body consists of over 250 different cell types and the epigenome is highly variable between these cell types^21^, various analyses of infant saliva cell composition were conducted. Previous research has shown that leukocytes and epithelial cells are both found in saliva samples that come from the oral cavities of children (mean age = 6.7 years)^22^, but to the best of our knowledge, no research has been done on infant saliva cellularity. To assess cellular composition, a small subsample of saliva samples (n = 8) from 6-week-old infants were examined under a microscope. The results showed platelet epithelial cells and bacteria, no immune cells, but as this was only a performed at 6 weeks on a small sample, leucocytes in the samples could not be excluded, the amount of leukocytes in all saliva samples was calculated using the leukocytes methylation for purity (LUMP) analysis^23^; the results showed that 42 of the 256 samples contained > 10% leukocytes. These DNA samples were excluded from the analyses. This choice of cutoff was made to exclude outliers, while keeping as may samples as possible. In total, 42 samples were removed, leaving 214 samples for the analyses.

### Computational analyses

Sociodemographic data analyses were performed using SPSS version 25 (IBM, SPSS Statistics, New York, NY, USA). The raw methylation data preprocessing was done with RnBeads v.1.2.1^24^ using the R programming language (http://www.r-project.org/). The methylation analysis was performed using MATLAB R2017B (The MathWorks Inc., Natick, MA, USA), and principal component analyses (PCA) were performed using Evince (Prediktera AB, Umeå, Sweden).

All statistical tests were done using two-sided student’s t-tests, assuming unequal variance, and any false discovery was corrected (q-value)^25^. Changes in differentially methylated regions (DMR) between 6 and 52 weeks were defined using the following criteria: q-value < 0.01, mean difference between groups > 0.2, and a minimum of two consecutive significant probes within a gene. All statistical analysis, t-distributed stochastic neighbor embedding (t-SNE), beta-histograms, sample scatter plots, and gene plots were generated using MATLAB version R2017b (The MathWorks, Inc., Natick, MA, USA). The PCA model was generated using Evince (Prediktera AB, Umeå, Sweden).

## Results

### Study population

The current study was the first to examine changes in DNA methylation between 6 and 52 weeks in infant saliva samples. The study population consisted of 153 Norwegian infants born mainly to mothers with a higher educational status and with stable living conditions^26^. The characteristic features of these infants at birth were as expected for infants born in Norway, except for the high maternal education status, which was overrepresented in this population^27^.

### Infant saliva samples show homogenous DNA methylation

**Figure 1:**
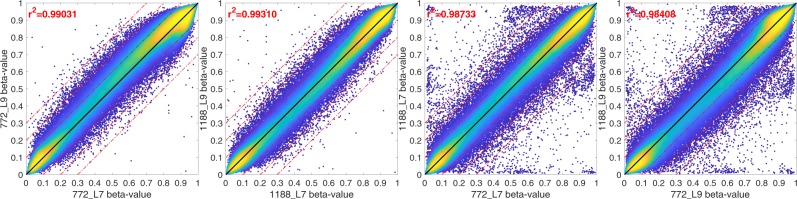
Sample scatter plots The sample-to-sample scatter plots display an anticipated cigar-like shape, indicating that the methylation levels for most probes are similar. The left two panels were generated using the same subject, with 6 weeks being displayed on the X-axis and 52 weeks on the Y-axis. The color indicates the probe density. These density plots show no large-scale methylation differences based on the time points, here with 6 weeks shown in blue and 52 weeks shown in red, but there is linear behavior, with most of the probes being located close to the diagonal. There is an increased number of probes that are differently methylated, that is, located far from the diagonal, indicating individual-specific methylation patterns that vary between the infants.

**Figure 2:**
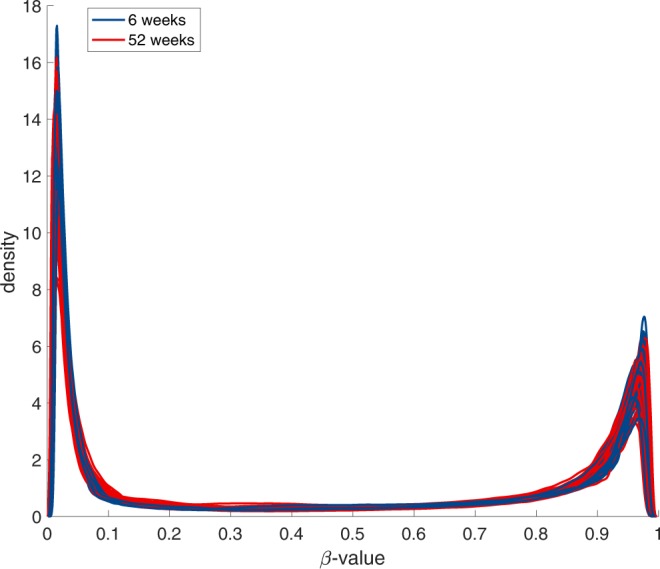
Beta value histogram The distribution of the beta values shows the expected bi-modal distribution, with the two peaks close to zero and one.

The DNA methylation QC analyses showed that there was minimal heterogeneity in methylation among the cell types assayed and that the methylation levels were more similar for the two time points for a single infant than between infants, as shown in Fig. 1, indicating that the samples were well suited for DNA methylation analyses. The distribution of the beta values across all the samples shows a clear bi-modal distribution, as shown in Fig. 2, which indicates minimal heterogeneity in methylation among the cell types assayed.

### Unsupervised dimensional reduction analyses show separation between time points

**Figure 3:**
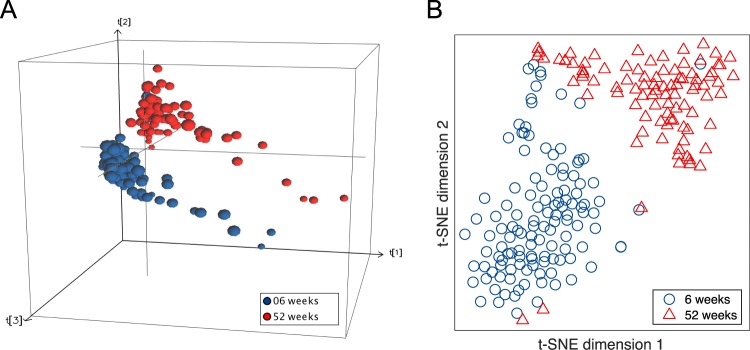
Unsupervised dimensionality reduction at separate time points The figure shows two different algorithms: (**A**) shows the results of the PCA, and (**B**) shows the t-SNE algorithm. Both show a clear separation between the 6-week samples (blue) and the 52-week samples (red) using all 423,315 probes.

The DNA methylation changes in infants between 6 and 52 weeks were analyzed using two separate algorithms (PCA) and t-distributed stochastic neighbor embedding (t-SNE), which are both agnostic to grouping issues. PCA is well-known algorithm used to investigate differences in data, while t-SNE is a newer, nonlinear dimensionality reduction algorithm that investigates the similarities in data^28^. The two different unsupervised dimensional reduction methods were applied to the samples using all 423,315 probes. Both algorithmic plots showed clear separations between the two time points, as shown in Fig. 3A,B. For PCA, the first three components showed clear separations between the two time points, as shown in Fig. 3A, and the algorithm gave similar results for t-SNE, as shown in Fig. 3B. These results indicate that there is a consistent time point difference and that this difference is greater than the individual differences.

### Statistically significant DNA methylation changes found in 42 genes

Stringent analytical criteria were selected to avoid discovering false DNA methylation sites because this was the first time that analyses have been performed to investigate DNA methylation changes for this population. Statistically significant DMRs were found in 42 genes in a total of 101 (out of 423,315) probes. The distribution of the probes within each gene showed consistent increases or decreases in methylation. A total of 36 genes showed increased methylation, and six showed decreased methylation. See Supplementary Table S1 for a detailed description of the results.

### Decreased DNA methylation was associated with only 6 genes

In total, six genes had decreased DNA methylation. *CLU*^29^ and *XAF1*^30^ are apoptosis inhibitors, *ORAOV1* regulates the cell cycle and apoptosis^31^, *PAQR7* regulates progesterone receptors^32^, and *EIF4E3* promotes messenger RNA transport and proliferation^33^, while *RTP4* has largely unknown functions.

### Increased DNA methylation associated with systemic biological processes

Change to ‘Of the 42 genes found to have statistically significant changes in DNA methylation, 13 genes that are associated with intracellular processes. The detailed results for these genes and their associated intracellular functions are presented in Table 2. Of the remaining genes, 24 were previously described as being associated with systemic biological functions 34 and all were found to have increased methylation. The genes and associated systemic functions are presented in Table 3’.

**Table 2:** Genes associated with intracellular processes.

Proposed function	Gene symbol	Direction	Location	Gene function associations	Epigenetic disease associations
Intracellular processes	*ARHGEF7 (β-PIX)*	+	Body	Cytoskeletal organization^61,62^	
Intracellular processes	*CLU*	−	TSS1500	Apoptosis inhibitor^29^	Alzheimer’s disease^63^, colon cancer^57^, and prostate cancer^58^
Intracellular processes	*NKX2–8*	+	3′UTR, TSS1500	Increases expression of *AFP*^64^	
Intracellular processes	*NRG2*	+	Body	Cell growth and differentiation^65^	Lung cancer^56^
Intracellular processes	*NXN*	+	Body	Cell growth and differentiation^66^	
Intracellular processes	*ORAOV1*	−	Body	Cell growth and apoptosis^31^	
Intracellular processes	*PAQR7*	−	Body	Progesterone receptor regulator^32^	
Intracellular processes	*RALB*	+	Body	Transmembrane signaling^67^	
Intracellular processes	*REC8*	+	1st exon, 5′UT	Chromosomal maintenance^68^	Gastric cancer^54^
					Thyroid cancer^55^
Intracellular processes	*XAF1*	−	1st exon, 5′UTR	Apoptosis inhibitor^30^	Lung cancer^53^
Long noncoding RNA	*C4orf19*	+	TSS200, 5′UTR	Long noncoding RNA	Colon cancer^51^
					Breast cancer^52^
Messenger RNA	*EIF4E3*	−	3′UTR	mRNA transport and proliferation^33^	
Messenger RNA	*MIR-135B*	+	TSS1500	Stability and translation of RNA	Cervical cancer^50^

^*^CpG sites that have been associated with DNA methylation changes in both the referred article and our research.

The table shows the genes found to have DNA methylation changes between 6 and 52 weeks, their proposed genetic function, their direction and location, their gene function, and their epigenetic disease associations. The location of the DNA methylation is marked as + for an increase and – for a decrease and can be found in one of the following regions: the promoter regions (TSS1500 and TSS200), 5′ untranslated region (UTR), 1st exon, gene body, and 3′UTR.

**Table 3:** Genes associated with systemic biological functions.

Proposed function	Gene symbol	Direction	Location	Gene function associations	Epigenetic disease associations
Gastrointestinal system					
Esophagus and stomach development	*BARX1*	+	Body	Transcription factor encoder^69^	Colon cancer^49^
Hematological system					
Erythrocyte production	*EPB49 (DMTN)*	+	5′UTR, TSS1500	Structural role in erythrocytes^70^	
Immune system					
Immune signaling pathway	*AFAP1*	+	Body	Immune response^71^	Barrett’s esophagus and esophageal adenocarcinoma^48^
Immune system regulation	*CYTH1*	+	Body	Adhesive properties^72^	
Lymphoid development	*IKZF4*	+	TSS1500,TSS200	Regulation of T cells^73^	
Metabolic system					
Drug metabolization and lipid synthesis	*CYP3A4*	+	TSS1500	Testosterone catalyst^74^	Liver metabolism^75^
Metabolism and lipid/cholesterol transport	*PLEKH8 (FAPP2)*	+	Body	Ciliary membrane formation^76^	Breast cancer and gliomas^47^
Weight	*PBX1*	+	Body	Osteogenesis^77,78^	Birth weight (cg06750897 and cg18181229)*^59^, obesity^79^, and acute leukemia^46^
Weight regulation	*KDM2B*	+	Body		Obesity (cg26995224 and cg13708645)*^79^,diabetes mellitus type II (cg13708645)*^80^ and colon cancer^45^
Musculoskeletal system					
Cranio-cervical joint development	*MEOX1*	+	TSS1500, 5′UTR, 1st exon	Somite development^81^	
Craniofacial development	*MN1*	+	3′UTR, Body	Cell proliferation^82^	Air pollution (cg20680669)*^83^
Muscle development	*TNNT3*	+	Body	Calcium regulation^84^	
Cardiac development	*SPEG*	+	Body	Myocyte development^85^	Environmental exposure (cg21117965)*^86^
Nervous system					
Reward system	*MAD1L1*	+	Body	Chromosome segregation^87^	
Neuronal regulation of energy balance	*MCHR1*	+	1st exon, Body	Energy homeostasis^88,89^	Schizophrenia (cg21342728)*^34^ and bipolar disorder^35^
Cerebral development	*NUAK1 (ARK5)*	+	TSS200, TSS1500	Cellular senescence and ploidy^90^	Suicide completers^91^
Cognition, behavior, and sleep regulation	*RAI1*	+	5′UTR	Neural tissue transcription^92,93^	Skeletal cancer (cg10140454)*^44^
Cognition	*S100B*	+	5′UTR	Calcium-binding protein secretion^94^	
Language development	*SEMA6D*	+	5′UTR	Signaling ligands^95^	
Executive function	*SLC1A2*	+	TSS1500	Glutamate clearance^96^	Neural function (cg25963980)*^37^, schizophrenia, and bipolar disorder^36^
Motor neuron and central nervous functions	*TBCD*	+	Body	Heterodimer assembly pathway^97^	Prenatal low glycemic diet (cg16538568)*^98^
Neural development	*TGM6*	+	TSS1500	Crosslinking of free amine group and glutamine^99^	Tic disorders (cg19391247)*^100^
Neural development	*MEIS2*	+	Body	Olfactory bulb neurogenesis and supraventricular neuroblasts^101^	Lung cancer^41^ and prostate cancer^42^
Visual system					
Eye development	*STRA6*	+	5′UTR,1st exon, TSS200	Regulation of retinol uptake in cells^102^	Endometriosis^103^
Eye development	*TEAD1*	+	Body	Regulation of growth and proliferation^43^	Colon cancer^43^

*CpG sites that have associated DNA methylation changes in both the referred article and our research.

The table shows the genes found to have DNA methylation changes between 6 and 52 weeks, their proposed genetic function, their direction and location, their gene function, and their epigenetic disease associations. The location of the DNA methylation is marked as + for an increase and – for a decrease and can be found in one of the following regions: the promoter regions (TSS1500 and TSS200), 5′ untranslated region (UTR), 1st exon, gene body, and 3′UTR.

#### MCHR1

**Figure 4:**
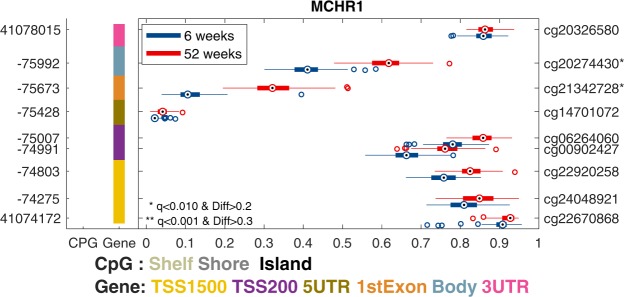
Methylation pattern by time point across multiple probes for *MCHR1* The graph shows the β-value on the X-axis across multiple probes for each gene. The left Y-axis shows the genomic coordinates for each probe, while the right Y-axis displays the probe Id. The CpG column to the right indicates the CpG island and the gene column in the gene body where the probe is from. For each probe, the methylation level is illustrated by a box plot, where the box is the interquartile range, and the median is the dot for the 6-week group (blue) and the 52-week group (red). Significant probes are shown with * for q < 0.01 and a difference > 0.2 and ** for q < 0.001 and difference > 0.3. The significant methylation probes for *MCHR1* are located in the 1st exon and in the body and are not found in a known CpG island.

The melanin-concentrating hormone receptor 1 (*MCHR1*) gene is an example of one of the genes with increased DNA methylation from 6 to 52 weeks. *MCHR1* has nine probes on the Illumina Infinium HumanMethylation450 BeadChip array. In these analyses, the results showed increased DNA methylation at two CpG sites: the 1st exon and the body. The mean DNA methylation increase for the two sites had an Δβ value of 0.21. DNA methylation changes in this gene have been associated with schizophrenia^34^ and bipolar disorder^35^. The changes in DNA methylation in the CpG site for the 1st exon, cg21342728, found in the current study, were described in the study of schizophrenia^34^. The DNA methylation changes for *MCHR1* are shown in Fig. 4.

#### SLC1A2

**Figure 5:**
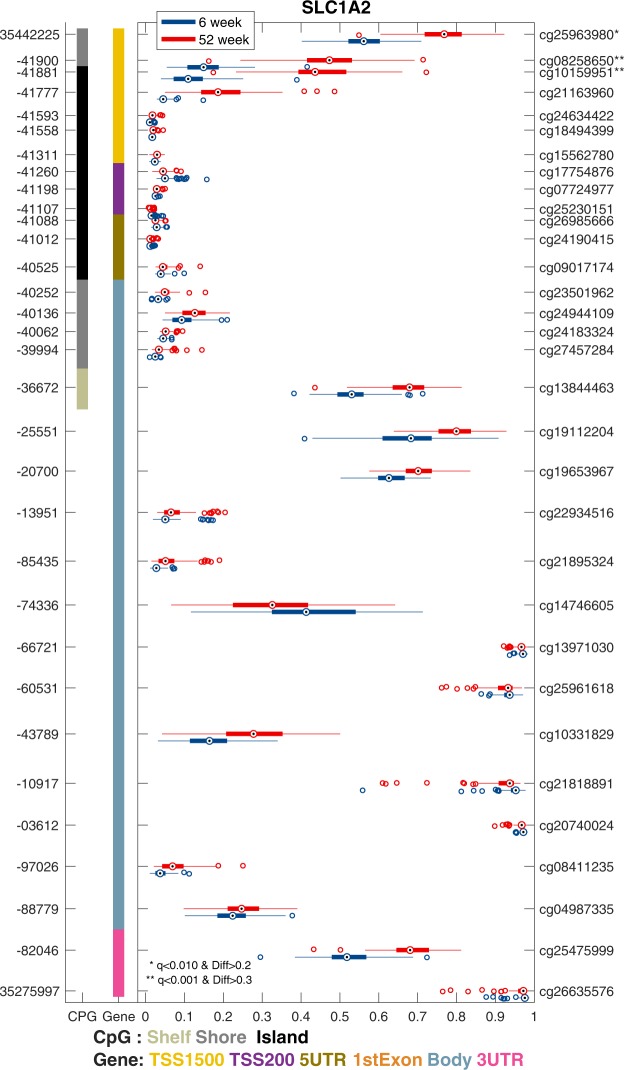
Methylation pattern by time point across multiple probes for *SLC1A2* The graph shows the β-value on the X-axis across multiple probes for each gene. The left Y-axis shows the genomic coordinates for each probe, while the right Y-axis displays the probe Id. The CpG column to the right indicates the CpG island and the gene column in the gene body where the probe is from. For each probe, the methylation level is illustrated by a box plot, where the box is the interquartile range, and the median is the dot for the 6-week group (blue) and the 52-week group (red). Significant probes are shown with * for q < 0.01 and difference > 0.2 and ** for q < 0.001 and difference > 0.3. The significant methylation probes for *SLC1A2* are located in the promoter area (TSS1500).

The solute carrier family 1 member 2 (*SLC1A2*) gene is another example of a gene with increased DNA methylation from 6 to 52 weeks. This gene has been found to have DNA methylation differences associated with schizophrenia and bipolar disorder^36^ and prematurity in infants ^37^. It has 32 probes on the Illumina Infinium HumanMethylation450 BeadChip array. The analyses showed a significant increase in methylation in the first three adjacent CpG sites in the promoter region (TSS1500) from 6 to 52 weeks. The mean DNA methylation increase of the three sites had an Δβ value of 0.29, and the largest difference was found at cg10159951, with a mean DNA methylation at 6 weeks of 0.12 and at 52 weeks of 0.45. The DNA methylation difference in the CpG site of the promoter island, cg25963980, has previously been associated with DNA methylation changes associated with infant prematurity ^37^. The DNA methylation changes for *SLC1A2* are shown in Fig. 5.

## Discussion

To the best of our knowledge, we conducted the first EWAS using Illumina450K analyses of infant saliva samples to study DNA methylation changes between 6 and 52 weeks of age. Two very different and unsupervised dimensionality algorithms—PCA and t-SNE—were used in the current study. The results showed clear separations between infant DNA methylation at the two studied time points. The greatest consistent difference is the time point and nonindividual differences. Because both the PCA and t-SNE showed the same separation in infant DNA methylation between the two time points, the findings indicate that the biological mechanisms associated with normal infant development in the first year of life are associated with DNA methylation changes.

To better understand which genes were associated with these changes, we analyzed the DNA methylation further and found that there were 42 genes across 101 probes with statistically significant DNA methylation changes. Of the 42 genes, 36 had increased DNA methylation, and six had decreased methylation levels. The effect of DNA methylation depends on where it occurs in the gene, and the DNA methylation changes in the current study occurred in different parts of the genes but most commonly were associated with decreased gene expression^17^.

The suggested decreased gene expression was unexpected as infants experience rapid developmental growth during the first year of life. However, infant development in the first year of life is relatively limited compared with the changes that occur during pregnancy^38^. The fetus develops from a single cell to a viable infant in 9 months, with an 875-million-fold increase in weight^39,40^; meanwhile, during the first year of life, the infant only experiences a three-fold increase in weight^2^. Therefore, although our findings are limited to buccal epithelial cells, they suggest a biological growth-slowing mechanism post-birth after the rapid fetal growth during pregnancy.

Our study has several strengths and limitations that need to be considered when interpreting the results. One important limitation for DNA methylation analyses is cell composition. It would have been better if the saliva sample at both 6 weeks and 52 weeks had been assessed. To compensate for this, LUMP scores were calculated for all saliva samples, finding leucocytes in the samples and using a cutoff of 10% for the analyses. However, if possible, further studies should consider assessing sample cell composition from all time points included in the analyses. Other limitations of the current study were that the population was made up of an overrepresentation of mothers with a high level of education compared with the general Norwegian population, and the study did not compare the characteristics of the whole “Little in Norway” cohort with the sample analyzed in the current study to assess selection bias and understand whether the findings are generalizable to the whole population.

One of the major strengths of the current study was that the saliva samples came from two time points, but still, these results cannot answer the question of whether DNA methylation fluctuates over the first year or if there is only an overall increase in DNA methylation. Therefore, to assess this, future studies should consider collecting more than two saliva samples over the first year of life. Another major strength was that the current study was based on the methodology used in cancer research because this field is at the forefront of epigenetic research, and human biology is the same for both cancer and biological development. Applying cutting-edge bioinformatical methods^28^ used in cancer research to examine biological mechanisms, 42 genes were found to have DNA methylation changes associated with early-life biological development. Of these, 14 had previously been found in different forms of cancers associated with epigenetic changes^41–58^, yet here, only two studies found epigenetic associations with development^37,59^. This might be because these genes are all associated with cancer, but it is just as likely that more cancer research is being conducted because of public and political efforts that increase cancer research funding.

The bioinformatic analysis of the epigenetic data commonly includes a determination of the significant differences at a single CpG site, considering them independently of each other and adjusting for false discovery rates^60^. Because this was the first study to investigate DNA methylation changes between 6 and 52 weeks, we wanted to avoid false positive results; therefore, we set stricter criteria for the significance of DMRs than what has been commonly used. If we had used other criteria, other significant DNA methylation changes would have been identified, but we wanted all of the discovered CpG sites to be correct and the probability of false positive findings to be small. Using this analytical model, we found 42 CpG sites with infant DNA methylation changes, but different analytical models for the analyses could reveal different results. Our choice of analytical method limited the possibility of correcting for confounders, such as gender. Future analyses should consider choosing a repeated measures model, so confounding variables can be adjusted for, this way confounding variables such as gender should be addressed. Hence, we encourage future studies of DNA methylation changes in infants to replicate the findings by using a repeated measures model and less stringent criteria to see if the same genes can be found, as well as investigating whether other genes are involved in development during this period.

## Conclusion

In conclusion, the algorithmic analyses showed that infant DNA methylation displays clear differences between 6 and 52 weeks. To investigate these differences further, two-sided student’s t-tests were performed. These analyses found 42 genes associated with DNA methylation changes. Of these, 36 genes showed increased and six decreased DNA methylation. The methylation changes indicated an overall decrease in gene expression, which, in turn, might represent a slowing mechanism to reduce the extensive growth development that occurs during pregnancy. Future studies of DNA methylation changes in infants could use repeated measures models and less stringent criteria to see if the same genes can be replicated, and whether other genes are involved in development during this period.

## Supplementary information

Supplementary information

## Data Availability

Data available upon request from vibeke.moe@psykologi.uio.no
