# Supplementary material for: DNA methylation changes in infants between 6 and 52 weeks
Source: Sci Rep. 2019 Nov 26;9:17587. doi: 10.1038/s41598-019-54355-z (PMC6879561; doi:10.1038/s41598-019-54355-z)
Supplement: Supplementary file 1 — Supplementary information [file 41598_2019_54355_MOESM1_ESM.pdf]

# Title page

# Supplementary information

# DNA methylation changes in infants between 6 and 52 weeks

Ellen Wikenius, Vibeke Moe, Lars Smith, Einar R. Heiervang, Anders Berglund

**Supplementary Table 1** includes detailed results from the gene analyses of the DNA methylation changes from 6 to 52 weeks based. The probe locations are based on the hg18 genome build.

Supplementary Table 1

| Gene | ProbeId | Chr | Position | q (t-test) | Δβ | Average 6 weeks | Average 52 weeks | Average Δβ |
|---|---|---|---|---|---|---|---|---|
| AFAP1 | cg20312457 | 4 | 7813475 | 3,23E-64 | 0,228 | 0,518 | 0,746 | |
| AFAP1 | cg02895699 | 4 | 7813791 | 2,63E-84 | 0,392 | 0,275 | 0,667 | |
| AFAP1 | cg18400079 | 4 | 7813911 | 1,06E-70 | 0,339 | 0,329 | 0,668 | 0,32 |
| ARHGEF7 | cg05472841 | 13 | 111932857 | 1,42E-45 | 0,251 | 0,330 | 0,581 | |
| ARHGEF7 | cg05420802 | 13 | 111932899 | 1,79E-31 | 0,238 | 0,604 | 0,842 | 0,24 |
| ATXN7L1 | cg19324118 | 7 | 105279494 | 1,27E-68 | 0,209 | 0,623 | 0,832 | |
| ATXN7L1 | cg27056270 | 7 | 105279549 | 8,22E-46 | 0,203 | 0,523 | 0,726 | |
| ATXN7L1 | cg24248505 | 7 | 105279790 | 6,84E-41 | 0,271 | 0,364 | 0,635 | 0,23 |
| BARX1 | cg13773631 | 9 | 96715843 | 2,48E-44 | 0,210 | 0,418 | 0,628 | |
| BARX1 | cg06071058 | 9 | 96716091 | 5,70E-43 | 0,215 | 0,469 | 0,684 | 0,21 |
| C4orf19 | cg20011360 | 4 | 37585660 | 1,70E-30 | 0,200 | 0,167 | 0,367 | |
| C4orf19 | cg04301614 | 4 | 37585993 | 2,93E-41 | 0,202 | 0,277 | 0,479 | 0,20 |
| CLU | cg22313574 | 8 | 27468981 | 5,07E-60 | -0,208 | 0,456 | 0,248 | |
| CLU | cg14917244 | 8 | 27469001 | 6,98E-73 | -0,296 | 0,498 | 0,201 | -0,25 |
| CYP3A4 | cg22821554 | 7 | 99383155 | 1,41E-62 | 0,268 | 0,237 | 0,505 | |
| CYP3A4 | cg09914773 | 7 | 99383224 | 2,62E-57 | 0,218 | 0,289 | 0,507 | 0,24 |
| CYTH1 | cg14653593 | 17 | 76676303 | 4,29E-64 | 0,211 | 0,441 | 0,653 | |
| CYTH1 | cg00472840 | 17 | 76676366 | 1,78E-67 | 0,207 | 0,580 | 0,787 | 0,21 |
| EIF4E3 | cg15037823 | 3 | 71730474 | 9,78E-79 | -0,292 | 0,512 | 0,221 | |
| EIF4E3 | cg01454951 | 3 | 71730677 | 7,90E-81 | -0,270 | 0,466 | 0,196 | -0,28 |
| EPB49 | cg03292675 | 8 | 21912864 | 3,66E-51 | 0,212 | 0,615 | 0,827 | |
| EPB49 | cg02046552 | 8 | 21914287 | 1,00E-57 | 0,287 | 0,567 | 0,854 | 0,25 |
| FBRSL1 | cg19771626 | 12 | 133127043 | 7,65E-55 | 0,203 | 0,259 | 0,462 | |
| FBRSL1 | cg00960772 | 12 | 133127270 | 1,81E-76 | 0,306 | 0,370 | 0,676 | 0,25 |
| IKZF4 | cg24414325 | 12 | 56414442 | 2,41E-82 | 0,310 | 0,347 | 0,657 | |
| IKZF4 | cg00026033 | 12 | 56414490 | 5,60E-96 | 0,219 | 0,281 | 0,501 | 0,26 |
| KDM2B | cg26995224 | 12 | 121974146 | 9,50E-64 | 0,203 | 0,193 | 0,396 | |
| KDM2B | cg13708645 | 12 | 121974305 | 1,67E-68 | 0,249 | 0,222 | 0,471 | 0,23 |
| MAD1L1 | cg14010305 | 7 | 2082961 | 5,93E-83 | 0,242 | 0,657 | 0,899 | |
| MAD1L1 | cg11792186 | 7 | 2083105 | 5,14E-76 | 0,276 | 0,404 | 0,680 | |
| MAD1L1 | cg15914316 | 7 | 2083197 | 2,71E-85 | 0,287 | 0,500 | 0,787 | |
| MAD1L1 | cg25352924 | 7 | 2083583 | 1,78E-55 | 0,222 | 0,422 | 0,644 | 0,26 |
| MCHR1 | cg21342728 | 22 | 41075673 | 3,47E-54 | 0,213 | 0,113 | 0,326 | |
| MCHR1 | cg20274430 | 22 | 41075992 | 1,78E-67 | 0,200 | 0,412 | 0,612 | 0,21 |
| MEIS2 | cg21579197 | 15 | 37350102 | 1,67E-59 | 0,221 | 0,652 | 0,873 | |
| MEIS2 | cg10451116 | 15 | 37350381 | 6,88E-49 | 0,202 | 0,458 | 0,660 | 0,21 |
| MEOX1 | cg04951104 | 17 | 41739195 | 1,74E-60 | 0,268 | 0,260 | 0,528 | |
| MEIS2 | cg22948288 | 17 | 41739246 | 2,10E-50 | 0,210 | 0,163 | 0,373 | 0,24 |

Supplementary Table 1

| | | | | | | | | |
|---|---|---|---|---|---|---|---|---|
| MIR135B | cg02520707 | 1 | 205418278 | 4,72E-56 | 0,287 | 0,197 | 0,484 | |
| MIR135B | cg13061767 | 1 | 205418410 | 7,89E-55 | 0,289 | 0,323 | 0,612 | 0,29 |
| MN1 | cg04959572 | 22 | 28145955 | 6,07E-65 | 0,230 | 0,208 | 0,438 | |
| MN1 | cg20680669 | 22 | 28189870 | 7,71E-71 | 0,414 | 0,343 | 0,757 | 0,32 |
| NKX2-8 | cg23705938 | 14 | 37049299 | 3,35E-94 | 0,323 | 0,403 | 0,726 | |
| NKX2-8 | cg23676577 | 14 | 37049565 | 4,03E-72 | 0,228 | 0,219 | 0,447 | |
| NKX2-8 | cg18396984 | 14 | 37049893 | 9,85E-51 | 0,277 | 0,070 | 0,347 | |
| NKX2-8 | cg06621744 | 14 | 37052470 | 3,47E-78 | 0,297 | 0,224 | 0,521 | |
| NKX2-8 | cg17929068 | 14 | 37052668 | 4,44E-69 | 0,236 | 0,398 | 0,634 | |
| NKX2-8 | cg26206196 | 14 | 37052771 | 7,90E-81 | 0,217 | 0,642 | 0,859 | |
| NKX2-8 | cg08081845 | 14 | 37053163 | 1,08E-61 | 0,232 | 0,199 | 0,431 | |
| NKX2-8 | cg20008148 | 14 | 37053169 | 1,32E-68 | 0,319 | 0,210 | 0,528 | 0,27 |
| NRG2 | cg05652757 | 5 | 139227606 | 1,22E-48 | 0,223 | 0,092 | 0,315 | |
| NRG2 | cg22060611 | 5 | 139227610 | 5,46E-52 | 0,216 | 0,063 | 0,279 | 0,22 |
| NUAK1 | cg17393917 | 12 | 106533840 | 1,85E-46 | 0,205 | 0,118 | 0,323 | |
| NUAK1 | cg23555120 | 12 | 106533863 | 5,30E-50 | 0,250 | 0,194 | 0,444 | |
| NUAK1 | cg26877596 | 12 | 106534217 | 2,44E-51 | 0,248 | 0,171 | 0,419 | |
| NUAK1 | cg05687834 | 12 | 106534276 | 7,62E-46 | 0,213 | 0,131 | 0,344 | 0,23 |
| NXN | cg16460383 | 17 | 818890 | 1,28E-45 | 0,207 | 0,080 | 0,287 | |
| NXN | cg09146872 | 17 | 820881 | 3,69E-44 | 0,215 | 0,631 | 0,846 | 0,21 |
| ORAOV1 | cg22206745 | 11 | 69484557 | 1,39E-70 | -0,265 | 0,453 | 0,187 | |
| ORAOV1 | cg03313574 | 11 | 69484574 | 4,43E-61 | -0,290 | 0,603 | 0,312 | -0,28 |
| PAQR7 | cg00465927 | 1 | 26190114 | 1,70E-61 | -0,211 | 0,665 | 0,454 | |
| PAQR7 | cg07551314 | 1 | 26190155 | 4,50E-50 | -0,239 | 0,924 | 0,685 | -0,22 |
| PBX1 | cg03323953 | 1 | 164544748 | 1,27E-55 | 0,296 | 0,341 | 0,638 | |
| PBX1 | cg06750897 | 1 | 164545553 | 6,72E-72 | 0,217 | 0,640 | 0,857 | |
| PBX1 | cg18181229 | 1 | 164545699 | 1,71E-58 | 0,213 | 0,303 | 0,516 | 0,24 |
| PLEKHA8 | cg08658009 | 7 | 30071179 | 1,55E-63 | 0,242 | 0,422 | 0,664 | |
| PLEKHA8 | cg15393925 | 7 | 30081299 | 6,09E-50 | 0,208 | 0,246 | 0,454 | 0,22 |
| RAI1 | cg27059698 | 17 | 17625521 | 1,33E-51 | 0,219 | 0,179 | 0,398 | |
| RAI1 | cg10140454 | 17 | 17626019 | 9,46E-44 | 0,228 | 0,488 | 0,717 | 0,22 |
| RALB | cg27505472 | 2 | 121036705 | 2,40E-59 | 0,252 | 0,343 | 0,595 | |
| RALB | cg25950520 | 2 | 121036760 | 3,17E-61 | 0,221 | 0,600 | 0,821 | 0,24 |
| REC8 | cg16473141 | 14 | 24641501 | 2,62E-58 | 0,226 | 0,169 | 0,395 | |
| REC8 | cg18512948 | 14 | 24641706 | 5,14E-57 | 0,236 | 0,125 | 0,361 | 0,23 |
| RTP4 | cg26824216 | 3 | 187086147 | 6,97E-58 | -0,225 | 0,350 | 0,125 | |
| RTP4 | cg15701237 | 3 | 187086154 | 3,00E-57 | -0,258 | 0,497 | 0,238 | -0,24 |
| S100B | cg11064537 | 21 | 48024639 | 1,32E-41 | 0,201 | 0,369 | 0,570 | |
| S100B | cg07152925 | 21 | 48024683 | 1,60E-50 | 0,218 | 0,292 | 0,510 | 0,21 |
| SEMA6D | cg09970175 | 15 | 48011443 | 1,05E-39 | 0,218 | 0,506 | 0,724 | |
| SEMA6D | cg19099049 | 15 | 48011466 | 1,87E-39 | 0,210 | 0,339 | 0,549 | 0,21 |

Supplementary Table 1

| | | | | | | | | |
|---|---|---|---|---|---|---|---|---|
| SLC1A2 | cg10159951 | 11 | 35441881 | 1,21E-56 | 0,334 | 0,116 | 0,450 | |
| SLC1A2 | cg08258650 | 11 | 35441900 | 7,85E-57 | 0,326 | 0,151 | 0,476 | |
| SLC1A2 | cg25963980 | 11 | 35442225 | 2,50E-51 | 0,201 | 0,564 | 0,765 | 0,29 |
| SPEG | cg21117965 | 2 | 220325369 | 8,24E-68 | 0,233 | 0,241 | 0,474 | |
| SPEG | cg07974504 | 2 | 220325375 | 6,00E-49 | 0,283 | 0,214 | 0,497 | 0,26 |
| STRA6 | cg26774156 | 15 | 74495384 | 3,69E-26 | 0,248 | 0,468 | 0,716 | |
| STRA6 | cg13888509 | 15 | 74495603 | 1,10E-40 | 0,208 | 0,531 | 0,739 | 0,23 |
| TBCD | cg01045697 | 17 | 80820757 | 8,30E-52 | 0,250 | 0,482 | 0,731 | |
| TBCD | cg08605840 | 17 | 80820763 | 9,67E-52 | 0,288 | 0,417 | 0,704 | |
| TBCD | cg16538568 | 17 | 80823310 | 1,61E-72 | 0,219 | 0,556 | 0,776 | |
| TBCD | cg23352492 | 17 | 80823397 | 2,90E-74 | 0,243 | 0,580 | 0,823 | 0,25 |
| TEAD1 | cg02703598 | 11 | 12831463 | 8,82E-26 | 0,213 | 0,407 | 0,620 | |
| TEAD1 | cg15573664 | 11 | 12833710 | 1,87E-35 | 0,249 | 0,377 | 0,626 | 0,23 |
| TGM6 | cg19391247 | 20 | 2360385 | 6,93E-48 | 0,225 | 0,437 | 0,662 | |
| TGM6 | cg06261066 | 20 | 2360396 | 4,98E-51 | 0,244 | 0,384 | 0,629 | 0,23 |
| TNNT3 | cg06679296 | 11 | 1949032 | 5,09E-69 | 0,264 | 0,583 | 0,847 | |
| TNNT3 | cg06503573 | 11 | 1949039 | 9,02E-67 | 0,247 | 0,653 | 0,900 | 0,26 |
| XAF1 | cg06085204 | 17 | 6659164 | 3,36E-57 | -0,260 | 0,519 | 0,259 | |
| XAF1 | cg05513208 | 17 | 6659189 | 2,51E-45 | -0,219 | 0,417 | 0,198 | |
| XAF1 | cg26502852 | 17 | 6659199 | 4,60E-66 | -0,265 | 0,398 | 0,133 | -0,25 |
| ZNF710 | cg19925872 | 15 | 90543392 | 2,39E-63 | 0,216 | 0,341 | 0,557 | |
| ZNF710 | cg23005885 | 15 | 90543450 | 1,25E-62 | 0,212 | 0,103 | 0,315 | 0,21 |
